# Predictors for clinical effectiveness of baricitinib in rheumatoid arthritis patients in routine clinical practice: data from a Japanese multicenter registry

**DOI:** 10.1038/s41598-020-78925-8

**Published:** 2020-12-14

**Authors:** Nobunori Takahashi, Shuji Asai, Tomonori Kobayakawa, Atsushi Kaneko, Tatsuo Watanabe, Takefumi Kato, Tsuyoshi Nishiume, Hisato Ishikawa, Yutaka Yoshioka, Yasuhide Kanayama, Tsuyoshi Watanabe, Yuji Hirano, Masahiro Hanabayashi, Yuichiro Yabe, Yutaka Yokota, Mochihito Suzuki, Yasumori Sobue, Kenya Terabe, Naoki Ishiguro, Toshihisa Kojima

**Affiliations:** 1 Department of Orthopedic Surgery and Rheumatology, Nagoya University Hospital, Nagoya University Graduate School of Medicine, 65 Tsurumai-cho, Showa-ku, Aichi, Nagoya 466-8550 Japan; 2 Kobayakawa Orthopedic and Rheumatologic Clinic, 1969 Kuno, Fukuroi, Shizuoka Japan; 3 Department of Orthopedic Surgery and Rheumatology, Nagoya Medical Center, 4-1-1 Sanno-maru, Naka-ku, Nagoya, Aichi Japan; 4 Department of Orthopedic Surgery, Daido Hospital, 9 Shiramizu-cho, Minami-ku, Nagoya, Aichi Japan; 5 Kato Orthopedic Clinic, 8-4 Minami-myoudaiji-cho, Okazaki, Japan; 6 Department of Rheumatology, Japanese Red Cross Nagoya Daiichi Hospital, 35 Michisita-cho, Nakamura-ku, Nagoya, Aichi Japan; 7 Department of Rheumatology, Handa City Hospital, 2-29 Toyo-cho, Handa, Aichi Japan; 8 Department of Orthopedic Surgery, Toyota Kosei Hospital, 500-1 Ibohara, Josui-cho, Toyota, Japan; 9 Department of Orthopedic Surgery, National Center for Geriatrics and Gerontology, 7-430 Morioka-cho, Obu, Aichi Japan; 10 Department of Rheumatology, Toyohashi Municipal Hospital, 50 Hakken-nishi, Aotake-cho, Toyohashi, Japan; 11 Department of Orthopedic Surgery, Ichinomiya Municipal Hospital, 2-2-22 Bunkyo, Ichinomiya, Japan; 12 Department of Rheumatology, Tokyo Shinjuku Medical Center, 5-1 Tsukudo-cho, Shinjuku-ku, Tokyo, Japan

**Keywords:** Rheumatoid arthritis, Outcomes research

## Abstract

This study aimed to evaluate the short-term effectiveness and safety profiles of baricitinib and explore factors associated with improved short-term effectiveness in patients with rheumatoid arthritis (RA) in clinical settings. A total of 113 consecutive RA patients who had been treated with baricitinib were registered in a Japanese multicenter registry and followed for at least 24 weeks. Mean age was 66.1 years, mean RA disease duration was 14.0 years, 71.1% had a history of use of biologics or JAK inhibitors (targeted DMARDs), and 48.3% and 40.0% were receiving concomitant methotrexate and oral prednisone, respectively. Mean DAS28-CRP significantly decreased from 3.55 at baseline to 2.32 at 24 weeks. At 24 weeks, 68.2% and 64.1% of patients achieved low disease activity (LDA) and moderate or good response, respectively. Multivariate logistic regression analysis revealed that no previous targeted DMARD use and lower DAS28-CRP score at baseline were independently associated with achievement of LDA at 24 weeks. While the effectiveness of baricitinib was similar regardless of whether patients had a history of only one or multiple targeted DMARDs use, patients with previous use of non-TNF inhibitors or JAK inhibitors showed lower rates of improvement in DAS28-CRP. The overall retention rate for baricitinib was 86.5% at 24 weeks, as estimated by Kaplan–Meier analysis. The discontinuation rate due to adverse events was 6.5% at 24 weeks. Baricitinib significantly improved RA disease activity in clinical practice. Baricitinib was significantly more effective when used as a first-line targeted DMARDs.

## Introduction

Rheumatoid arthritis (RA) is a chronic autoimmune inflammatory disease characterized by persistent synovitis and joint destruction. Conventional synthetic disease modifying anti-rheumatic drugs (csDMARDs), such as methotrexate (MTX), are first-line drugs usually prescribed for the treatment of RA^1,2^. Three classes of biological DMARDs (bDMARDs) are also used in patients with inadequate response or intolerance to csDMARDs. Both csDMARDs and bDMARDs show clinical effectiveness with acceptable safety in a substantial proportion of RA patients. However, agents with novel mechanisms of action are always in demand, as a consistent proportion of patients are refractory or intolerant to existing agents.

Janus kinase (JAK) inhibitors have recently been added as a novel treatment option for RA^3^. JAK is a family of intracellular, non-receptor tyrosine kinases that transduce cytokine-mediated signals via the JAK-signal transducer and activator of transcription (STAT) pathway. Inhibition of the JAK-STAT pathway has been demonstrated to be an effective strategy for treating several diseases including RA^4,5^. The JAK family has four members: JAK1, JAK2, JAK3, and tyrosine kinase 2 (Tyk2). Each cytokine receptor has a specific combination of JAK family members at its intracellular domain, e.g., JAK1/2 for interferon γ, JAK1/2 and Tyk2 for IL-6, and double JAK2 for erythropoietin receptor^6^.

Baricitinib, a selective inhibitor of JAK signaling^7,8^, is considered a specific JAK1/2 inhibitor, as it has similar inhibitory potency against JAK1 and JAK2 but is much less potent against JAK3 and Tyk2^9^. A number of randomized controlled trials (RCTs) have reported on the clinical efficacy and safety profile of baricitinib in RA patients^7,10,11^. In particular, baricitinib had a significantly superior ACR20 response rate at 12 weeks compared to adalimumab, an anti-TNF agent, in RA patients who showed inadequate response to MTX^12^. While some multi-national RCTs have examined the clinical outcomes of Japanese patients with RA who were treated with baricitinib^13–15^, clinical data for Japanese RA patients in routine clinical practice are scarce.

In Japan, three classes of bDMARDs have been available in the clinical practice of RA (anti-TNF since 2013, anti-IL-6R since 2008, and CTLA4-Ig since 2011)^16–18^. In addition to the bDMARDs, tofacitinib a first JAK inhibitor has been available since 2013. Baricitinib was launched as the second JAK inhibitor in 2017.

In this study, we used data from a Japanese multicenter registry system to investigate the clinical effectiveness and safety profile of baricitinib for 24 weeks. Specifically, we examined changes in lymphocyte count, which has been shown to decrease by treatment with tofacitinib, another JAK inhibitor^19^, changes in hemoglobin levels, which is expected to decrease via inhibition of erythropoietin signaling^6^, and the incidence of herpes zoster, as treatment with JAK inhibitors including baricitinib has been reported to increase the risk of herpes zoster in a Japanese sub-population^13^.

## Materials and methods

### Participants

All eligible patients were registered in and followed by the Tsurumai Biologics Communication Registry (TBCR), a registry of patients with RA starting treatment with biologics or tsDMARDs (targeted DMARDs), which was developed for the purpose of analyzing the long-term prognosis of RA biologic treatment in clinical practice^20^. Data were collected prospectively from 2008, as well as retrospectively for patients who had been treated with biologics up until 2008. All 2827 patients registered in the TBCR as of April 2015 met the 1987 American College of Rheumatology (ACR) or the 2010 ACR/ European League Against Rheumatism (EULAR) classification criteria for RA^21^. Information on medication history was collected during clinic visits to TBCR-affiliated institutions. Registry data are updated once per year and include information on drug continuation, reasons for discontinuation (e.g., insufficient effectiveness), and adverse events (AEs). Patient anonymity was maintained during data collection, and security of personal information was strictly controlled. This study was approved by the ethics committees of Nagoya University Graduate School of Medicine and TBCR-affiliated institutions. Written informed consent was obtained from all participants of this study. All methods were carried out in accordance with relevant guidelines and regulations.

The present study included 113 consecutive patients treated with baricitinib who were prospectively observed for longer than 24 weeks at TBCR-affiliated institutions. Baricitinib has been commercially available for RA treatment since 2017; therefore, data in this study were all prospectively collected. Patients took 2 or 4 mg baricitinib once a day, according to the drug label and Japan College of Rheumatology guidelines for treatment.

### Data collection

The following demographic data were recorded at the initiation of treatment (baseline, week 0): age, sex, disease duration, Krebs von den Lungen-6 (KL-6)^22^, estimated glomerular filtration rate (eGFR), lymphocyte count, hemoglobin levels, radiological structural damage of joints (Steinbrocker stage), daily dysfunction (Steinbrocker class), anti-cyclic citrullinated peptide antibody (ACPA) positivity (normal limit [NL] < 4.5 U/mL), rheumatoid factor (RF) positivity (NL < 20 IU/mL), history and number of previous biologics or JAK inhibitors (targeted DMARDs), and concomitant treatment [MTX and prednisolone (PSL)]. The following disease parameters were recorded at baseline and after 4, 12, 24, and 52 weeks of treatment: tender joint count (TJC) and swollen joint count (SJC) on 28 joints, patient’s (Pt) and physician’s (Ph) global assessment (GA) of disease activity, modified health assessment questionnaire (mHAQ) score^23,24^, serum C-reactive protein (CRP) levels, erythrocyte sedimentation rate (ESR), and matrix metalloproteinase-3 (MMP-3) levels. Disease activity was evaluated at each time point using the 28-joint disease activity score with CRP (DAS28-CRP), which includes data from the above-mentioned disease parameters.

### Disease activity and EULAR response

The DAS28-CRP is known to significantly underestimate disease activity and overestimate improvement in disease activity compared to the DAS28-erythrocyte sedimentation rate (ESR)^25^. Therefore, in the present study, we used criteria that differed from those of DAS28-ESR not to overestimate the effectiveness of baricitinib. Disease activity was categorized as follows: DAS28-CRP remission (REM; DAS28-CRP < 2.3), LDA (2.3 ≤ DAS28-CRP < 2.7), moderate disease activity (MDA; 2.7 ≤ DAS28-CRP ≤ 4.1), and high disease activity (HDA; DAS28-CRP > 4.1). These criteria have been validated in a large Japanese cohort study^26^.

Disease activity was evaluated at baseline and after 4, 12, 24, and 52 weeks of treatment. The response to baricitinib therapy at 4, 12, and 24 weeks were evaluated by the EULAR response criteria using 4.1 and 2.7 as the thresholds for the HDA and LDA, respectively^26^.

### Statistical analysis

Demographic and disease characteristics were evaluated using descriptive statistics. All results are expressed as mean ± standard deviation (SD) or percentage (%). Student’s t test was used for 2-group comparisons, and the chi-square test for categorical variables. Differences among three groups were analyzed by analysis of variance (ANOVA) for continuous variables. Significance of individual differences was evaluated with the Bonferroni test if ANOVA was significant. The last observation carried forward (LOCF) method was used in each analysis^27^.

Kaplan–Meier curves were generated to estimate rates of continuation and discontinuation due to insufficient efficacy and AEs. The log-rank test was used for comparisons of continuation and discontinuation rates among groups^27^.

Multivariate logistic regression analysis was performed to determine predictive factors of LDA achievement at week 24. Variables significantly associated with the endpoint in univariate analysis (*p* < 0.05), as well as age and sex, and a stepwise selection process were used to select the final model. Adjusted odds ratios (ORs) with 95% confidence intervals (CIs) were calculated^28^.

All statistical tests were two-sided, and significance was defined as *p* < 0.05. All analyses were performed with SPSS version 22 software (IBM Corp., Armonk, NY, USA).

## Results

### Demographic data

Baseline characteristics of the 113 patients enrolled in this study are shown in Table 1. Patients were predominantly female (79.3%), mean age was 66.1 years, and mean disease duration was 14.0 years. Most patients were seropositive for ACPA (82.1%) and RF (77.1%). More than half of patients (71.1%) had a history of previous targeted DMARDs use, with a mean number of targeted DMARDs used being 2.1. Less than half of the patients received concomitant MTX or PSL.

**Table 1:** Patient baseline characteristics.

N	113
Age (years)	66.1 ± 12.8
Sex (% female)	79.3
BMI (kg/m^2^)	22.7 ± 3.8
Disease duration (year)	14.0 ± 14.0
Stage (i/ii/iii/iv, %)	18.2/37.2/17.4/27.3
Class (i/ii/iii/iv, %)	17.4/57.0/24.8/0.8
ACPA positive (%)	82.1
RF positive (%)	77.1
KL-6 (U/mL)	338.7 ± 318.8
eGFR (mL/min/1.73 m^2^)	78.5 ± 26.9
Lymph (/µL)	1447 ± 759
Hb (g/dL)	11.9 ± 1.6
Previous targeted DMARDs (%)	71.1
Number of previous targeted DMARDs^a^	2.1 ± 1.3
MTX use (%)	48.3
MTX dose (mg/week)^a^	10.1 ± 3.0
Oral prednisolone use (%)	40.0
Oral prednisolone dose (mg/day)^a^	3.9 ± 2.0
DAS28-CRP	3.52 ± 1.20
TJC, 0–28	3.2 ± 3.9
SJC, 0–28	3.2 ± 3.6
PtGA, 0–100 mm	42.9 ± 28.2
CRP (mg/dL)	1.5 ± 2.2
ESR (mm/h)	39.8 ± 30.4
MMP-3 (ng/mL)	205.3 ± 276.3
PhGA, 0–100 mm	35.3 ± 23.8
mHAQ	0.67 ± 0.63

Data are presented as mean ± standard deviation unless otherwise indicated.

*BMI* Body mass index, *Stage* Steinbrocker’s stage, *Class* Steinbrocker’s class, *ACPA* anti-citrullinated peptide antibody, *RF* rheumatoid factor, *KL-6* Krebs von den Lungen-6, *eGFR* estimated glomerular filtration rate, *Lymph* lymphocyte count, *Hb* hemoglobin level, *targeted DMARDs* biological or targeted synthetic disease-modifying antirheumatic drugs, *MTX* methotrexate, *DAS28* Disease Activity Score in 28 joints, *TJC* tender joint count, *SJC* swollen joint count, *PtGA* patient global assessment, *CRP *C-reactive protein, *ESR* erythrocyte sedimentation rate, *MMP-3* matrix metalloproteinase-3, *PhGA* physician’s global assessment, *mHAQ* modified health assessment questionnaire.

^a^Mean among patients receiving the drug.

### Changes in disease activity

**Figure 1:**
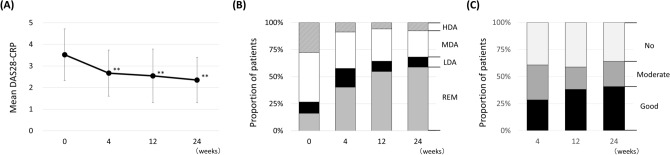
Overall clinical effectiveness of baricitinib for 24 weeks in rheumatoid arthritis patients. (**A**) Mean Disease Activity Score in 28 joints using C-reactive protein (DAS28-CRP) score. (**B**) Patient categorical distribution of disease activity based on DAS28-CRP score. (**C**) Distribution of the European League Against Rheumatism (EULAR) response achievement rate. **p < 0.01 in paired Student’s *t* test, compared to baseline.

Mean DAS28-CRP score was 3.55 ± 1.21 at baseline and significantly decreased to 2.65 ± 1.06 at 4 weeks and 2.32 ± 1.03 at 24 weeks (Fig. 1A). The categorical distribution of disease activity also improved from baseline to 24 weeks (Fig. 1B). The proportion of patients who achieved LDA significantly increased from 26.7 to 68.2% (p < 0.001), and the proportion of those who achieved remission also significantly increased from 16.2% to 58.9% (p < 0.001). The proportion of patients who achieved moderate or good response was 60.8% at 4 weeks and 64.1% at 24 weeks, and the proportion of those who achieved good EULAR response was 28.4% at 4 weeks, increasing to 40.8% at 24 weeks (Fig. 1C). No significant difference was observed in EULAR response rate between 4 and 24 weeks.

Mean MMP-3 value was 205.3 ± 276.3 at baseline and significantly decreased to 150.5 ± 282.7 at 4 weeks (p < 0.01), 170.7 ± 303.8 at 12 weeks (p < 0.01), and 156.3 ± 291.4 at 24 weeks (p < 0.01).

### Changes in usage rate and dose of concomitant MTX and PSL

Proportions of MTX users were similar between baseline (48.2%) and 24 weeks (46.4%). Doses of concomitant MTX significantly decreased from baseline (9.9 ± 3.2 mg/week) to 24 weeks (9.5 ± 3.2 mg/week) (p = 0.031). Proportions of PSL users slightly decreased from baseline (40.2%) to 24 weeks (34.0%) (p = 0.359). Doses of PSL significantly decreased from baseline (3.9 ± 2.1 mg/week) to 4 weeks (3.6 ± 2.0 mg/week) (p = 0.010) and 24 weeks (3.2 ± 2.1 mg/week) (p = 0.006).

### Rates of treatment retention and discontinuation due to inadequate response and AEs

**Figure 2:**
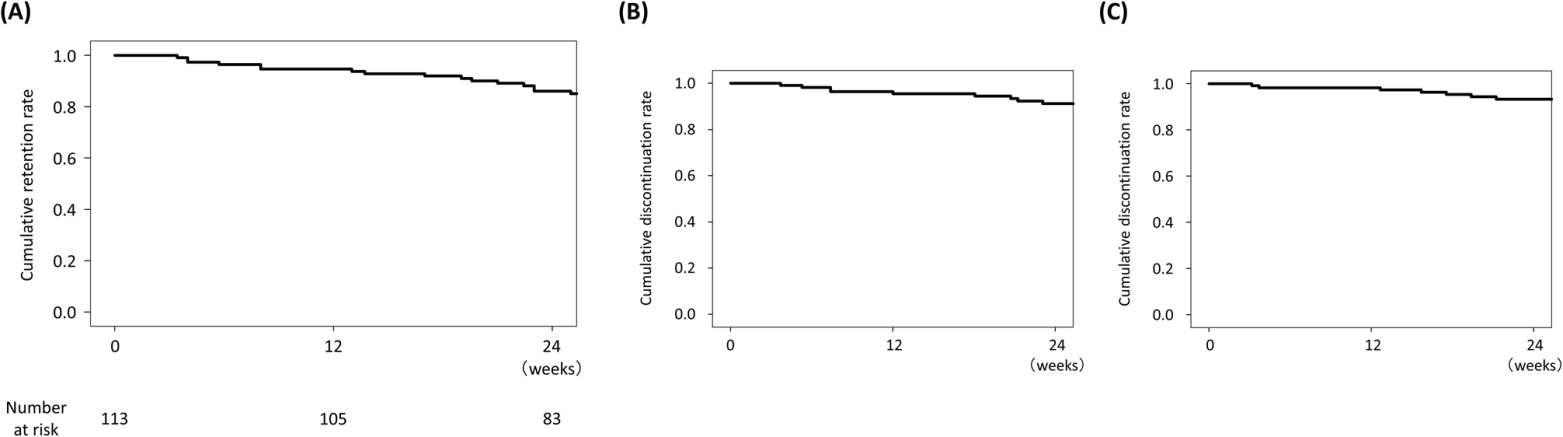
Kaplan–Meier curves for time to discontinuation of baricitinib. (**A**) Overall drug retention rate. (**B**) Drug retention rate with discontinuation due to inadequate response as the endpoint. (**C**) Drug retention rate with discontinuation due to adverse events as the endpoint.

The retention rate was 86.5% at 24 weeks, as estimated by Kaplan–Meier analysis (Fig. 2A). The most frequent reason for discontinuation was inadequate response to baricitinib (7.4% at 24 weeks, Fig. 2B). Six patients discontinued baricitinib due to AEs within 24 weeks. The rate of discontinuation due to AEs was 6.5% at 24 weeks (Fig. 2C). The observed AEs included interstitial pneumonia in 2 patients, renal dysfunction in 2 patients, pneumonia in 1 patient, and malignant mesothelioma in 1 patient.

### Changes in lymphocyte counts and hemoglobin levels

Lymphocyte counts and hemoglobin levels were examined, as decrease in these values may occur as major AEs during treatment with JAK inhibitors. Contrary to this assumption, however, no decrease in lymphocyte counts or hemoglobin levels was observed (Figure S1). Mean lymphocyte counts significantly increased from 1458 ± 767 at baseline to 1662 ± 884 at 4 weeks (p < 0.001) and 1677 ± 748 at 24 weeks (p < 0.001). No significant differences were observed in hemoglobin levels between baseline (11.8 ± 1.6) and 24 weeks (11.7 ± 1.7) (p = 0.394).

### Incidence of herpes zoster

The incidence of herpes zoster, another major AE associated with JAK inhibitor treatment, was also examined. The incidence rate was calculated as the number of unique patients with an event per 100 patient-years (P-Y) of observation time. In the present study cohort, seven patients developed herpes zoster, with an incidence rate of 8.4 per 100 P-Y. There was no patient that developed severe herpes zoster. Mean duration to herpes zoster development since baricitinib started was 22.1 weeks. Mean age of the seven patients was 70.7 years, mean disease duration of RA was 11.4 years, mean BMI was 22.9 (kg/m^2^), and mean eGFR was 73.6 (mL/min/1.73 m^2^). Proportion of patients that concomitantly used MTX and PSL was 57.1% and 42.9%, respectively (Table S2). All seven patients were treated with antiviral agents for herpes zoster and restarted baricitinib treatment.

### Factors predicting achievement of LDA at 24 weeks

Univariate and multivariate logistic regression analyses were performed to identify predictors of LDA achievement at 24 weeks. In the univariate logistic regression analysis, the following variables were found to be associated with LDA achievement at 24 weeks after baricitinib initiation: no targeted DMARDs use, concomitant PSL use, mHAQ score at baseline, and DAS28-CRP score at baseline (Table 2). The multivariate logistic regression analysis revealed no targeted DMARDs use and lower DAS28-CRP score at baseline to be independently associated with LDA achievement at 24 weeks.

**Table 2:** Predictive factors for LDA achievement at week 24 (univariate and multivariate logistic regression analyses).

Variables	Univariate		Multivariate	
	OR (95% CI)	p-value	OR (95% CI)	p-value
Male	1.17 (0.43–3.16)	0.755		
Age, < 65 years	1.46 (0.62–3.44)	0.388		
Disease duration, < 10 years	1.41 (0.61–3.23)	0.419		
ACPA, > 4.5	1.57 (0.51–4.80)	0.433		
No previous targeted DMARDs use	4.67 (1.49–14.66)	0.008	33.40 (2.53–442.62)	0.008
Concomitant MTX	0.86 (0.40–2.02)	0.789		
Concomitant PSL	0.24 (0.10–0.56)	0.001		
DAS28-CRP at baseline	0.55 (0.38–0.80)	0.002	0.28 (0.13–0.62)	0.002
mHAQ at baseline	0.27 (0.09–0.77)	0.015		

*ACPA* anti-citrullinated peptide antibody, *targeted DMARDs* biological or targeted synthetic disease-modifying antirheumatic drugs, *MTX* methotrexate, *PSL* prednisolone, *DAS28* Disease Activity Score in 28 joints, *mHAQ* modified health assessment questionnaire.

We also compared the background characteristics between the patients with MDA or lower disease activity and those with still HDA at 24 weeks. The patients that demonstrated HDA even after 24 weeks of baricitinib treatment had significantly higher disease activity at baseline (Table S1). This result would be consistent with the result of multivariate logistic regression analysis mentioned above.

### Comparisons of change in DAS28-CRP between patients with and without concomitant MTX use

**Figure 3:**
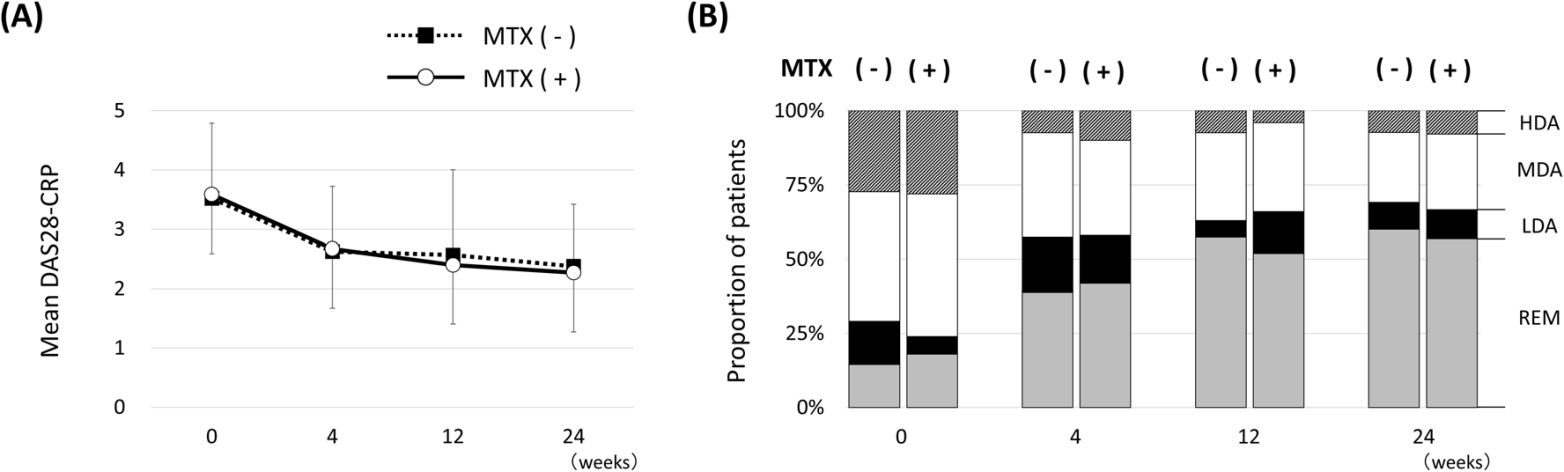
Comparisons of Disease Activity Score based on 28 joints (DAS28-CRP) between patients with and without concomitant methotrexate (MTX) use. (**A**) Mean and standard deviation (SD) for DAS28-CRP. (**B**) Categorical distribution of DAS28-CRP in patients with and without concomitant MTX use. Disease activity was categorized as follows: remission (REM; DAS28-CRP < 2.3), low disease activity (LDA; 2.3 ≤ DAS28-CRP < 2.7), moderate disease activity (MDA; 2.7 ≤ DAS28-CRP ≤ 4.1), and high disease activity (HDA; DAS28-CRP > 4.1).

Concomitant MTX use was not associated with LDA achievement at 24 weeks in the logistic regression analysis. We then presumed that there might be differences in responsiveness to baricitinib at earlier timing. We compared mean DAS28-CRP scores at baseline, 4, 12, and 12 weeks between patients with and without concomitant MTX use (Fig. 3A). No significant difference was observed between the two groups in DAS28-CRP at each time point. We also examined the categorical distribution of DAS28-CRP (Fig. 3B) and found no significant difference in proportions of patients who achieved LDA or REM at each time point between the two groups.

### Comparisons of DAS28-CRP between patients with and without previous targeted DMARDs use

**Figure 4:**
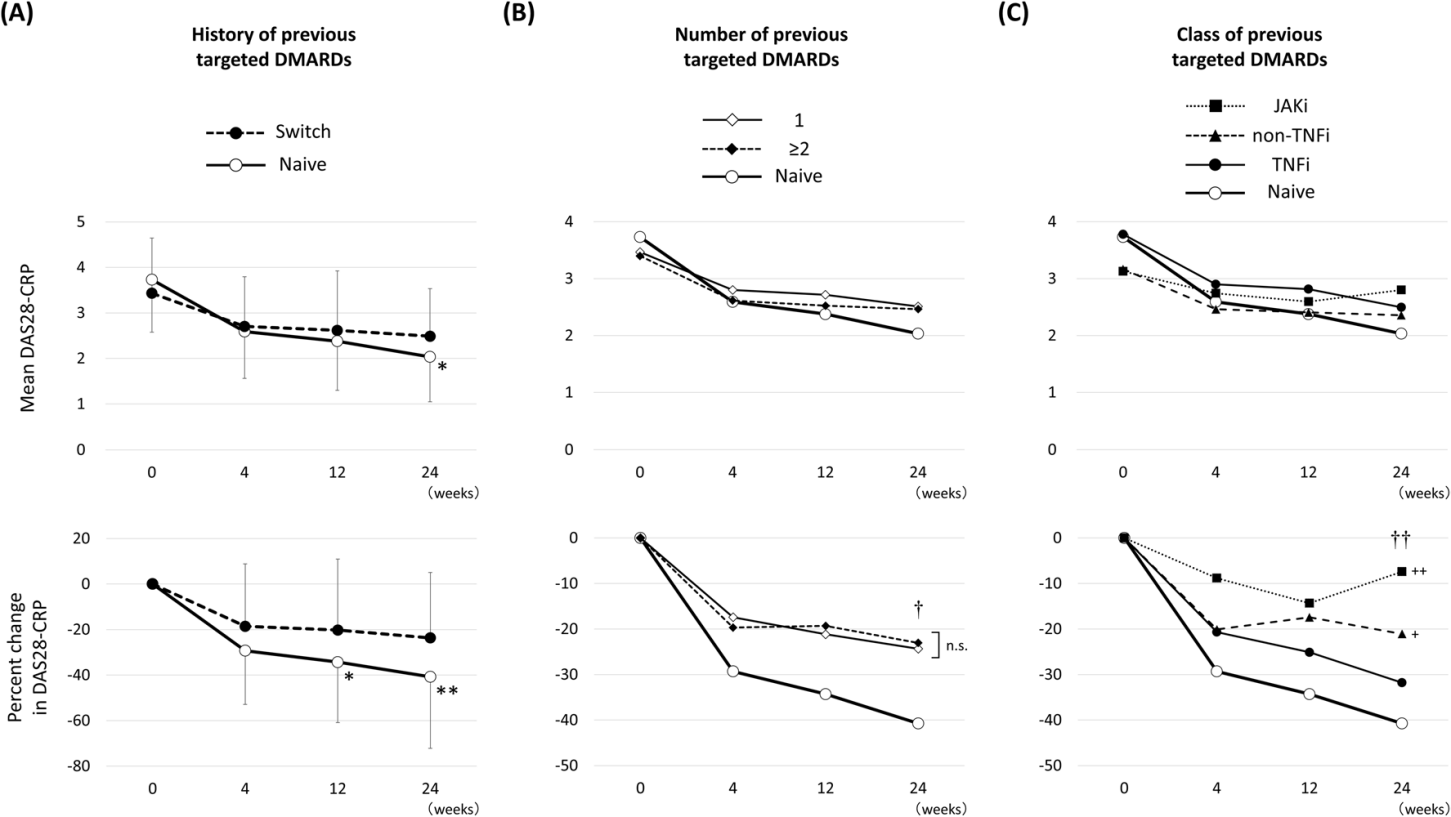
Mean and percent change in Disease Activity Score based on 28 joints (DAS28-CRP). (**A**) Comparisons between patients with (Switch) and without (Naïve) previous targeted DMARDs (biological or targeted synthetic DMARDs). *p < 0.05, **p < 0.01 in paired Student’s *t* test, compared to baseline. (**B**) Comparisons between naïve, patients with only 1, and patients with ≥ 2 previous targeted DMARDs. ^†^p < 0.05 in one-way ANOVA. *n.s.* not significant in post-hoc Bonferroni test. (**C**) Comparisons between patients without (Naïve) and with previous tumor necrosis factor inhibitors (TNFi), non-TNFi (IL-6 receptor inhibitors or abatacept), and Janus kinase inhibitors (JAKi). ^††^p < 0.01 in one-way ANOVA. ^+^p < 0.05, ^++^p < 0.01 in post-hoc Bonferroni test, compared to naïve.

No previous targeted DMARDs use was significantly associated with LDA achievement at 24 weeks in the logistic regression analysis. Mean DAS28-CRP at 24 weeks was significantly lower in the patients without previous targeted DMARDs use (Naïve, N = 32) compared to that in the patients with previous targeted DMARDs (Switch, N = 81) (2.04 ± 0.99 vs 2.49 ± 1.04, p = 0.032) (Fig. 4A, upper graph). The comparison of percent change from baseline in DAS28-CRP between the Naïve and Switch groups revealed significant differences at 12 weeks (− 34.3 ± 26.6 vs − 20.2 ± 31.1, p = 0.025) and 24 weeks (− 40.7 ± 31.5 vs − 23.7 ± 28.7, p = 0.006) (Fig. 4A lower graph).

Next, we subdivided the Switch group into two groups according to whether patients used only one previous targeted DMARDs (N = 38) or more than two (N = 43). No significant differences were observed in mean DAS28-CRP and percent change in DAS28-CRP at each time point between the two groups (Fig. 4B).

We then subdivided the Switch group into three groups according to the class of previous targeted DMARDs: TNF inhibitors [TNFi; N = 38 (7 infliximab, 16 etanercept, 1 adalimumab, 12 golimumab, and 2 certolizumab pegol)], non-TNFi [N = 31 (20 tocilizumab, 1 sarilumab, and 10 abatacept)], and JAK inhibitors [JAKi; N = 12 (11 tofacitinib and 1 upadacitinib)]. A subsequent post-hoc Bonferroni test revealed a significant difference between the non-TNFi (− 21.1 ± 30.3, p = 0.044) and Naïve group (− 40.7 ± 31.5) and between the JAKi (− 13.7 ± 9.8, p = 0.005) and Naïve group, but not between the TNFi (− 31.8 ± 26.0, p = 1.00) and Naïve group (Fig. 4C).

## Discussion

In this study, we demonstrated the short-term clinical effectiveness and safety profile of baricitinib in Japanese RA patients in the ‘real-world’ setting. Baricitinib significantly improved disease activity, with an expected safety profile. We observed some interesting features regarding the effectiveness of baricitinib. To the best of our knowledge, this study is the first to report the clinical outcomes of baricitinib in routine clinical practice in Japan.

This study included patients with background of relatively old, long disease duration, and low MTX combination rate. Major proportion of patients experienced average of two previous targeted DMARDs treatments. In other words, the patients included in this study had very different backgrounds compared with those in the clinical trials. We presumed that baricitinib was sometimes used like a last resort after trying various treatments in the tolerated elderly patients with safety items in the normal range such as KL-6 and eGFR in the clinical practice in Japan. The primary value of this study is that we demonstrated the effectiveness and safety of baricitinib in the ‘real world’ patients with diverse background.

The effectiveness of baricitinib was not associated with concomitant MTX use. We typically use MTX as the first-line csDMARD in clinical practice. However, some RA patients are intolerant to MTX and thus are instead treated with other csDMARDs and/or non-TNF inhibitors such as anti-IL6R agents and CTLA4-Ig, which have shown reasonable effectiveness^29–31^. The RA-BEGIN study, which was conducted in RA patents with no or limited prior DMARD treatment, had the following three arms: MTX alone, baricitinib alone, and combination^11^. The ACR 20% response rate, i.e., the primary endpoint, in the baricitinib alone group (76.7%) was similar to that of the combination group (78.1%). These findings were consistent with the results obtained in the present study. Although no RCT has compared baricitinib with and without concomitant MTX in RA patients, our results suggest the usefulness of baricitinib as a viable treatment option for patients who are not treated with MTX.

The effectiveness of baricitinib was significantly superior in the targeted DMARDs-naïve patients like the results of some previous targeted DMARDs. We previously reported the similar result in the patients treated with abatacept using data from the same registry^32^. Data from Japanese post-marketing surveillance demonstrated biologics-naïve patients achieved better clinical effectiveness at 24 weeks in adalimumab, tocilizumab, and abatacept^16–18^. No previous reports have previously addressed this point in baricitinib. Two clinical trials, the RA-BEAM study and the RA-BUILD study, which were conducted in bDMARD-naïve RA patients with inadequate response (IR) to MTX and other csDMARDs, respectively, have reported an ACR20 response rate at 12 weeks of 69.6% and 65.9%, respectively^10,12^. Another study, the RA-BEACON study, targeting bDMARD-IR patients reported an ACR20 response rate at 12 weeks of 55.4%^8^, which was slightly lower than that in the RA-BEAM or RA-BUILD. Although these rates cannot be directly compared due to different patient backgrounds among the three clinical trials, considering the current data, baricitinib appears to demonstrate higher effectiveness when used as a first-line targeted DMARDs.

Interestingly, the rate of improvement in DAS28-CRP in patients previously treated with non-TNFi or JAKi tended to be low. The subgroup analysis of the RA-BEACON study reported no significant consistent interactions for ACR20 by the number of prior bDMARDs, TNFi, or non-TNFi^33^. This report is consistent with our findings regarding the number of prior bDMARDs. RA-BEACON study evaluated the percentage of patients achieving CDAI ≤ 10 at 24 weeks in subgroups defined by previous use of bDMARDs, and calculated the odds ratio with 95% confidence interval (CI) for baricitinib versus placebo. The range of CI apparently crossed the 1.0 line of no significance in the subgroup with previous use of non-TNFi compared with placebo. In the current study, we found significant difference only in the percent change from baseline in DAS28-CRP shown in the Fig. 4C. Difference in mean DAS28-CRP at baseline, if not significant, may have affected this result. Although a further study is necessary, the effectiveness of baricitinib may be reduced in patients previously treated with non-TNFi or JAKi.

There was no significant decrease in lymphocyte count in RA patients treated with BAR for 24 weeks. Interestingly, our clinical practice data show a significant increase in lymphocyte count at week 4. A previous report described the safety profile of baricitinib, including changes in lymphocyte count using an integrated database, which included data from eight phase III/II/Ib clinical trials and one long-term extension study. Lymphocyte counts were increased at 2 weeks and gradually decreased to the baseline level at 24 weeks in one clinical trial^34^. Our data are consistent with those from these clinical trials.

Hemoglobin levels did not significantly decrease either. Since the erythropoietin signal is transduced via JAK2^6^, baricitinib, a JAK1/2 inhibitor, was expected to reduce serum hemoglobin levels. Indeed, in clinical trials, a small decrease in hemoglobin levels relative to baseline has been reported^3,34^. On the other hand, active inflammation in RA itself can induce chronic anemia, which is mediated by hepcidin as part of the acute-phase response^35^. We assumed that hemoglobin levels were consistent during baricitinib treatment because of the balanced relationship between the increase due to anti-inflammation and the decrease due to JAK2 inhibition.

Seven patients developed herpes zoster during the observation period. The incidence rate of herpes zoster has been reported to be higher in the Japanese population compared to international populations^15^. The incidence rate of herpes zoster was 8.4 per 100 P-Y, which was slightly higher than the previously reported rate of 6.5 per 100 P-Y among the Japanese population in baricitinib clinical trials^15^. Old age is reportedly a risk factor for herpes zoster in the Japanese population^36^. Another report demonstrated that the concomitant glucocorticoid but not methotrexate increased the risk for herpes zoster in tofacitinib-treated RA patients^37^. The mean age of patients in the present study was 66.1 years and was higher compared to that reported in the clinical trials (53.9 years)^15^. Careful observation is necessary especially when elderly patients are treated with baricitinib. Repeated patient education regarding the risk of herpes zoster and immediate medication are important for preventing or decreasing the severity of herpes zoster^38^.

Mean doses of concomitant MTX and PSL were slightly decreased from baseline to 24 weeks in this study. These data suggest that the improvement of disease activity in this study were not due to the effects of dose increasing of these drugs, and at the same time the baricitinib treatment may reduce the dose of these drugs.

The present study has several limitations. First, data regarding the concomitant use of csDMARDs other than MTX, which could have affected the clinical efficacy of baricitinib, were not available. Second, sequential radiographic data were not available. Given the importance of joint protective effects in demonstrating clinical efficacy, evaluating radiographic changes in patients treated with baricitinib will be necessary in the future. Third, the observation period was too short to obtain a robust conclusion regarding the long-term effectiveness and safety analysis in the present study. Fourth, only a few patients were included in this study. Using data from a larger number of patients, some other predictive factors for effectiveness of baricitinib may be found in the future analysis. Fifth, this study has no comparison group. We cannot discuss how good or bad was the effectiveness and safety profile of baricitinib compared to other biologics or JAK inhibitors. Sixth, our registry system has not been collecting data regarding smoking status of patients treated with baricitinib. Since the smoking status has been known to affect the disease activity of RA^39,40^, it may have affected the results in this study.

## Conclusion

Baricitinib showed significant effectiveness in Japanese RA patients in routine clinical practice, with an expected safety profile. Baricitinib was significantly more effective when used as a first-line targeted DMARDs and may play a key role in the modern treatment strategy for RA, although careful observation is necessary for possible complications and AEs including herpes zoster.

## Supplementary Information

Supplementary Figure S1.

Supplementary Table S1.

Supplementary Table S2.
