# Supplementary material for: Predictors for clinical effectiveness of baricitinib in rheumatoid arthritis patients in routine clinical practice: data from a Japanese multicenter registry
Source: Sci Rep. 2020 Dec 14;10:21907. doi: 10.1038/s41598-020-78925-8 (PMC7736589; doi:10.1038/s41598-020-78925-8)
Supplement: Supplementary file 1 — Supplementary Figure S1. [file 41598_2020_78925_MOESM1_ESM.docx]

**Original Research Article**

**Predictors for clinical effectiveness of baricitinib in rheumatoid arthritis patients in routine clinical practice: Data from a Japanese multicenter registry**

Nobunori Takahashi^1^, Shuji Asai^1^, Tomonori Kobayakawa^2^, Atsushi Kaneko^3^, Tatsuo Watanabe^4^, Takefumi Kato^5^, Tsuyoshi Nishiume^1^, Hisato Ishikawa^6^, Yutaka Yoshioka^7^, Yasuhide Kanayama^8^, Tsuyoshi Watanabe^9^, Yuji Hirano^10^, Masahiro Hanabayashi^11^, Yuichiro Yabe^12^, Yutaka Yokota^1^, Mochihito Suzuki^1^, Yasumori Sobue^1^, Kenya Terabe^1^, Naoki Ishiguro^1^, and Toshihisa Kojima^1^

1. Department of Orthopedic Surgery and Rheumatology, Nagoya University Graduate School of Medicine, 65 Tsuruma-cho, Showa-ku, Nagoya, Aichi, Japan
2. Kobayakawa Orthopedic and Rheumatologic Clinic, 1969 Kuno, Fukuroi, Shizuoka, Japan

(3) Department of Orthopedic Surgery and Rheumatology, Nagoya Medical Center, 4-1-1 Sanno-maru, Naka-ku, Nagoya, Aichi, Japan

(4) Department of Orthopedic Surgery, Daido Hospital, 9 Shiramizu-cho, Minami-ku, Nagoya, Aichi, Japan

(5) Kato Orthopedic Clinic, 8-4 Minami-myoudaiji-cho, Okazaki, Japan

(6) Department of Rheumatology, Japanese Red Cross Nagoya Daiichi Hospital, 35 Michisita-cho, Nakamura-ku, Nagoya, Aichi, Japan

(7) Department of Rheumatology, Handa City Hospital, 2-29 Toyo-cho, Handa, Aichi, Japan

(8) Department of Orthopedic Surgery, Toyota Kosei Hospital, 500-1 Ibohara, Josui-cho, Toyota, Japan

(9) Department of Orthopedic Surgery, National Center for Geriatrics and Gerontology, 7-430 Morioka-cho, Obu, Aichi, Japan

(10) Department of Rheumatology, Toyohashi Municipal Hospital, 50 Hakken-nishi, Aotake-cho, Toyohashi, Japan

(11) Department of Orthopedic Surgery, Ichinomiya Municipal Hospital, 2-2-22 Bunkyo, Ichinomiya, Japan

(12) Department of Rheumatology, Tokyo Shinjuku Medical Center, 5-1 Tsukudo-cho, Shinjuku-ku, Tokyo, Japan


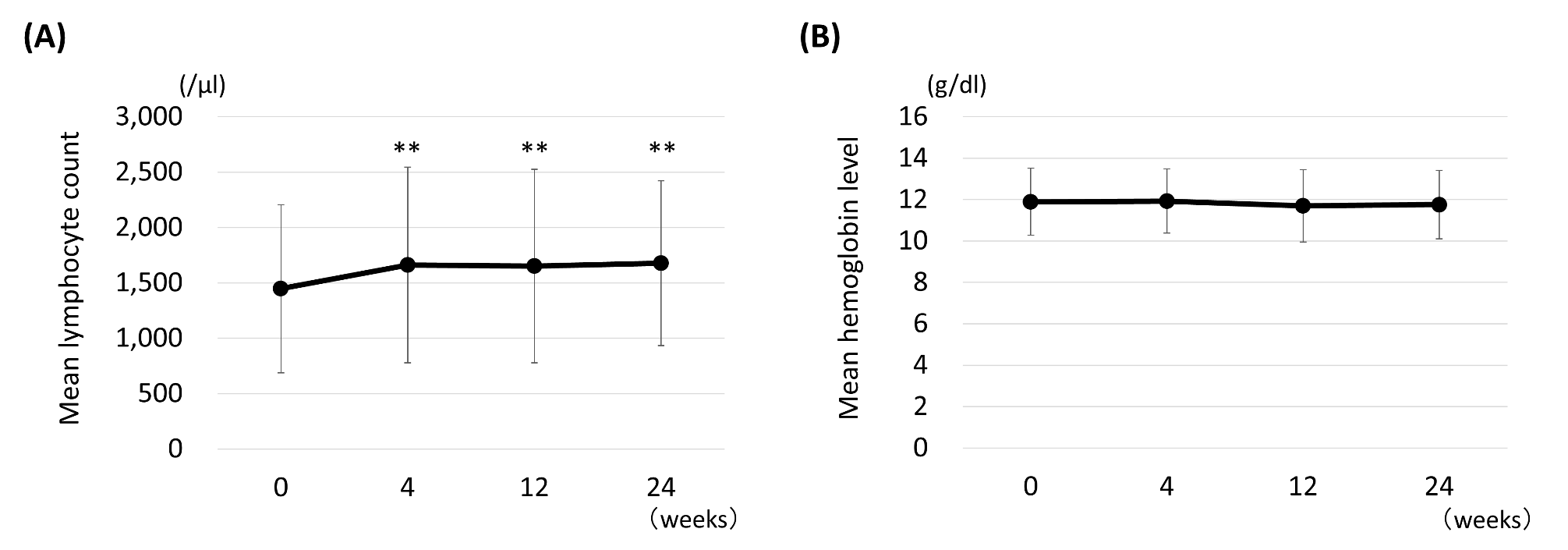


**Figure S1.** Evaluation of safety endpoints. (A) Change in lymphocyte count. (B) Change in hemoglobin levels. ** p < 0.01 in paired student t-test, compared to baseline.
