# Supplementary material for: Predictors for clinical effectiveness of baricitinib in rheumatoid arthritis patients in routine clinical practice: data from a Japanese multicenter registry
Source: Sci Rep. 2020 Dec 14;10:21907. doi: 10.1038/s41598-020-78925-8 (PMC7736589; doi:10.1038/s41598-020-78925-8)
Supplement: Supplementary file 2 — Supplementary Table S1. [file 41598_2020_78925_MOESM2_ESM.docx]

**Original Research Article**

**Predictors for clinical effectiveness of baricitinib in rheumatoid arthritis patients in routine clinical practice: Data from a Japanese multicenter registry**

Nobunori Takahashi^1^, Shuji Asai^1^, Tomonori Kobayakawa^2^, Atsushi Kaneko^3^, Tatsuo Watanabe^4^, Takefumi Kato^5^, Tsuyoshi Nishiume^1^, Hisato Ishikawa^6^, Yutaka Yoshioka^7^, Yasuhide Kanayama^8^, Tsuyoshi Watanabe^9^, Yuji Hirano^10^, Masahiro Hanabayashi^11^, Yuichiro Yabe^12^, Yutaka Yokota^1^, Mochihito Suzuki^1^, Yasumori Sobue^1^, Kenya Terabe^1^, Naoki Ishiguro^1^, and Toshihisa Kojima^1^

1. Department of Orthopedic Surgery and Rheumatology, Nagoya University Graduate School of Medicine, 65 Tsuruma-cho, Showa-ku, Nagoya, Aichi, Japan
2. Kobayakawa Orthopedic and Rheumatologic Clinic, 1969 Kuno, Fukuroi, Shizuoka, Japan

(3) Department of Orthopedic Surgery and Rheumatology, Nagoya Medical Center, 4-1-1 Sanno-maru, Naka-ku, Nagoya, Aichi, Japan

(4) Department of Orthopedic Surgery, Daido Hospital, 9 Shiramizu-cho, Minami-ku, Nagoya, Aichi, Japan

(5) Kato Orthopedic Clinic, 8-4 Minami-myoudaiji-cho, Okazaki, Japan

(6) Department of Rheumatology, Japanese Red Cross Nagoya Daiichi Hospital, 35 Michisita-cho, Nakamura-ku, Nagoya, Aichi, Japan

(7) Department of Rheumatology, Handa City Hospital, 2-29 Toyo-cho, Handa, Aichi, Japan

(8) Department of Orthopedic Surgery, Toyota Kosei Hospital, 500-1 Ibohara, Josui-cho, Toyota, Japan

(9) Department of Orthopedic Surgery, National Center for Geriatrics and Gerontology, 7-430 Morioka-cho, Obu, Aichi, Japan

(10) Department of Rheumatology, Toyohashi Municipal Hospital, 50 Hakken-nishi, Aotake-cho, Toyohashi, Japan

(11) Department of Orthopedic Surgery, Ichinomiya Municipal Hospital, 2-2-22 Bunkyo, Ichinomiya, Japan

(12) Department of Rheumatology, Tokyo Shinjuku Medical Center, 5-1 Tsukudo-cho, Shinjuku-ku, Tokyo, Japan

Table 1. Patient baseline characteristics

|  | MDA or lower  at 24 weeks | HDA  at 24 weeks |  |
| --- | --- | --- | --- |
| N | 105 | 8 | p-value |
| Age (year) | 66.7 ± 12.2 | 62.0 ± 18.1 | 0.313 |
| Gender (% female) | 76.8 | 87.5 | 0.484 |
| BMI (kg/m^2^) | 22.6 ± 3.6 | 23.1 ± 3.6 | 0.729 |
| Disease duration (year) | 13.5 ± 14.5 | 20.0 ± 11.9 | 0.218 |
| Stage (i/ii/iii/iv,%) | 19.2/37.4/18.2/25.3 | 12.5/37.5/25.0/25.0 | 0.948 |
| Class (i/ii/iii/iv,%) | 20.2/60.6/19.2/0.0 | 0.0/25.0/75.0/0.0 | ***0.001*** |
| ACPA positive (%) | 84.8 | 71.4 | 0.355 |
| RF positive (%) | 78.6 | 66.7 | 0.502 |
| KL-6 (U/ml) | 329.1 ± 330.5 | 317.3 ± 208.7 | 0.926 |
| eGFR (ml/min/1.73m^2^) | 77.0 ± 24.1 | 86.3 ± 51.0 | 0.624 |
| Lymph (/µl) | 1443 ± 773 | 1119 ± 414 | 0.244 |
| Hb (g/dL) | 11.9 ± 1.6 | 10.9 ± 1.4 | 0.087 |
| Previous bDMARDS (%) | 69.7 | 75.0 | 0.753 |
| Number of previous bDMARDs^a^ | 2.0 ± 1.2 | 2.5 ± 2.3 | 0.607 |
| MTX use (%) | 48.0 | 50.0 | 0.912 |
| MTX dose (mg/week)^a^ | 10.0 ± 3.1 | 8.0 ± 2.8 | 0.214 |
| Oral prednisolone use (%) | 36.7 | 62.5 | 0.150 |
| Oral prednisolone dose (mg/day)^a^ | 3.5 ± 2.1 | 5.9 ± 1.2 | ***0.017*** |
| DAS28-CRP | 3.45 ± 1.12 | 5.07 ± 1.21 | ***<0.001*** |
| TJC,0-28 | 3.0 ± 4.0 | 6.6 ± 3.5 | ***0.015*** |
| SJC,0-28 | 3.1 ± 3.6 | 7.0 ± 3.6 | ***0.005*** |
| PtGA, 0-100 mm | 42.2 ± 27.2 | 60.3 ± 37.6 | 0.083 |
| CRP (mg/dL) | 1.3 ± 1.8 | 4.9 ± 4.9 | 0.082 |
| ESR (mm/h) | 38.4 ± 29.9 | 51.1 ± 31.4 | 0.291 |
| MMP-3 (ng/mL) | 182.1 ± 161.0 | 273.0 ± 214.7 | 0.168 |
| PhGA, 0-100 mm | 34.5 ± 22.9 | 53.1 ± 29.1 | ***0.033*** |
| mHAQ | 0.65 ± 0.58 | 0.94 ± 1.21 | 0.671 |

Data are presented as mean ± standard deviation unless otherwise indicated.

MDA: moderate disease activity based on DAS28-CRP score, HDA: high disease activity based on DAS28-CRP score, BMI: Body mass index, Stage: Steinbrocker’s stage, Class: Steinbrocker’s class, ACPA: anti-citrullinated peptide antibody, RF: rheumatoid factor, KL-6: Krebs von den Lungen-6, eGFR: estimated glomerular filtration rate, Lymph: lymphocyte count, Hb: hemoglobin level, targeted DMARDs: biological or targeted synthetic disease-modifying antirheumatic drugs, MTX: methotrexate, DAS28: Disease Activity Score in 28 joints, TJC: tender joint count, SJC: swollen joint count, PtGA: patient global assessment, CRP: C-reactive protein, ESR: erythrocyte sedimentation rate, MMP-3: matrix metalloproteinase-3, PhGA: physician’s global assessment, mHAQ: modified health assessment questionnaire

^a^ Mean among patients receiving the drug

***Bold italic***: p < 0.05
