# Supplementary material for: Predictors for clinical effectiveness of baricitinib in rheumatoid arthritis patients in routine clinical practice: data from a Japanese multicenter registry
Source: Sci Rep. 2020 Dec 14;10:21907. doi: 10.1038/s41598-020-78925-8 (PMC7736589; doi:10.1038/s41598-020-78925-8)
Supplement: Supplementary file 3 — Supplementary Table S2. [file 41598_2020_78925_MOESM3_ESM.docx]

**Original Research Article**

**Predictors for clinical effectiveness of baricitinib in rheumatoid arthritis patients in routine clinical practice: Data from a Japanese multicenter registry**

Nobunori Takahashi^1^, Shuji Asai^1^, Tomonori Kobayakawa^2^, Atsushi Kaneko^3^, Tatsuo Watanabe^4^, Takefumi Kato^5^, Tsuyoshi Nishiume^1^, Hisato Ishikawa^6^, Yutaka Yoshioka^7^, Yasuhide Kanayama^8^, Tsuyoshi Watanabe^9^, Yuji Hirano^10^, Masahiro Hanabayashi^11^, Yuichiro Yabe^12^, Yutaka Yokota^1^, Mochihito Suzuki^1^, Yasumori Sobue^1^, Kenya Terabe^1^, Naoki Ishiguro^1^, and Toshihisa Kojima^1^

1. Department of Orthopedic Surgery and Rheumatology, Nagoya University Graduate School of Medicine, 65 Tsuruma-cho, Showa-ku, Nagoya, Aichi, Japan
2. Kobayakawa Orthopedic and Rheumatologic Clinic, 1969 Kuno, Fukuroi, Shizuoka, Japan

(3) Department of Orthopedic Surgery and Rheumatology, Nagoya Medical Center, 4-1-1 Sanno-maru, Naka-ku, Nagoya, Aichi, Japan

(4) Department of Orthopedic Surgery, Daido Hospital, 9 Shiramizu-cho, Minami-ku, Nagoya, Aichi, Japan

(5) Kato Orthopedic Clinic, 8-4 Minami-myoudaiji-cho, Okazaki, Japan

(6) Department of Rheumatology, Japanese Red Cross Nagoya Daiichi Hospital, 35 Michisita-cho, Nakamura-ku, Nagoya, Aichi, Japan

(7) Department of Rheumatology, Handa City Hospital, 2-29 Toyo-cho, Handa, Aichi, Japan

(8) Department of Orthopedic Surgery, Toyota Kosei Hospital, 500-1 Ibohara, Josui-cho, Toyota, Japan

(9) Department of Orthopedic Surgery, National Center for Geriatrics and Gerontology, 7-430 Morioka-cho, Obu, Aichi, Japan

(10) Department of Rheumatology, Toyohashi Municipal Hospital, 50 Hakken-nishi, Aotake-cho, Toyohashi, Japan

(11) Department of Orthopedic Surgery, Ichinomiya Municipal Hospital, 2-2-22 Bunkyo, Ichinomiya, Japan

(12) Department of Rheumatology, Tokyo Shinjuku Medical Center, 5-1 Tsukudo-cho, Shinjuku-ku, Tokyo, Japan

Table S2. Characteristics of seven patients that developed herpes zoster

| Case | Time since BAR started | Sex | Age | Disease duration | BMI | eGFR | MTX dose | PSL dose | Temporary interruption of BAR | Treatment with  antivirals |
| --- | --- | --- | --- | --- | --- | --- | --- | --- | --- | --- |
|  | (weeks) |  | (years) | (years) | (kg/m^2^) | (ml/min/1.73 m^2^) | (mg/weeks) | (mg/day) |  |  |
| 1 | 4.0 | Female | 85 | 4.0 | 21.2 | 40.1 | 0 | 5.0 | Yes | Yes |
| 2 | 5.0 | Female | 76 | 19.9 | 22.8 | 41.4 | 6 | 0.5 | Yes | Yes |
| 3 | 8.3 | Female | 68 | 11.4 | 20.6 | 102.3 | 12 | 0.0 | Yes | Yes |
| 4 | 21.6 | Female | 68 | 11.1 | 23.6 | 130.6 | 0 | 0.0 | Yes | Yes |
| 5 | 22.6 | Male | 68 | 9.4 | 22.1 | 71.8 | 0 | 0.0 | Yes | Yes |
| 6 | 37.0 | Female | 59 | 4.7 | 28.0 | 55.3 | 10 | 0.0 | Yes | Yes |
| 7 | 56.3 | Female | 71 | 19.0 | 21.9 | 73.8 | 12 | 2.0 | No | Yes |

BAR: baricitinib, BMI: Body mass index, eGFR: estimated glomerular filtration rate, MTX: methotrexate, PSL: prednisolone
